# Prevalence of abdominal aortic aneurysm and associated lower extremity artery aneurysm in men hospitalized for suspected or known cardiopulmonary disease

**DOI:** 10.1186/s12872-019-1265-2

**Published:** 2019-12-09

**Authors:** Anna Hohneck, Michael Keese, Gerhard Ruemenapf, Klaus Amendt, Hannelore Muertz, Katharina Janda, Ibrahim Akin, Martin Borggrefe, Martin Sigl

**Affiliations:** 1 First Department of Medicine, University Medical Centre Mannheim, Medical Faculty Mannheim, University of Heidelberg, Mannheim, Germany; 2 DZHK (German Centre for Cardiovascular Research), partner site Mannheim, Mannheim, Germany; 3 Department of Vascular Surgery, University Medical Centre Mannheim, Medical Faculty Mannheim, University of Heidelberg, Mannheim, Germany; 4 Department of Vascular Surgery, Diakonissen-Stiftungs-Krankenhaus Speyer, Speyer, Germany; 5 Department of Angiology, Cardiology and Diabetes associated diseases, Diakonissenkrankenhaus Mannheim, Gefäßzentrum Oberrhein, Mannheim, Germany

**Keywords:** Abdominal aortic aneurysm, Screening, High-risk cohort, Lower extremity artery aneurysm

## Abstract

**Background:**

AAA is a disease affecting predominantly male patients ≥65 years and its dreaded complications such as rupture led to population-based screening programs as preventive measure. Nonetheless, the supposed prevalence may have been overestimated, so that targeted screening of high risk populations may be more effective.

This study was performed to evaluate the prevalence of abdominal aortic aneurysm (AAA) of an inpatient high-risk cohort and to estimate the co-prevalence of lower extremity arterial aneurysms.

**Methods:**

Participants**:** 566 male inpatients, ≥ 65 years of age, hospitalized for suspected or known cardiopulmonary disease.

Primary and secondary outcome measures: Maximal infrarenal aortic diameters using abdominal ultrasound (leading edge to leading edge method). Upon detection of an AAA (diameter ≥ 30 mm), the lower extremity arteries were examined with regard to associated aneurysms.

**Results:**

In 40 of 566 patients (7.1%) AAAs were detectable. Fourteen patients (2.5%) had a first diagnosis of AAA, none of which was large (> 55 mm), the remaining 26 patients were either already diagnosed (14 patients, 2.5%) or previously repaired (12 patients, 2.1%).

The three most common main diagnoses at discharge were acute coronary syndrome (43.3%), congestive heart failure (32.2%), and chronic obstructive pulmonary disease (12%). The cohort showed a distinct cardiovascular risk profile comprising arterial hypertension (82.9%), diabetes mellitus (44.4%), and a history of smoking (57.6%).

In multivariate analysis, three-vessel coronary artery disease (Odds ratio (OR): 4.5, 95% confidence interval (CI): 2.3–8.9, *p* <  0.0001) and history of smoking (OR: 3.7, CI: 1.6–8.6, *p* <  0.01) were positively associated with AAA, while diabetes mellitus (OR: 0.5, CI: 0.2–0.9, *p* = 0.0295) showed a negative association with AAA.

Among the subjects with AAA, we found two large iliac and two large popliteal aneurysms.

**Conclusion:**

Ultrasound screening in male inpatients, hospitalized for suspected or known cardiopulmonary disease, revealed a high AAA prevalence in comparison to the present epidemiological screening programs. There was a moderate proportion of newly-screen detected AAA and additional screening of the lower extremity arteries yielded some associated aneurysms with indication for possible intervention.

## Background

Implementation of national screening programs of aortic abdominal aneurysms (AAA) in European countries and in the United States of America (USA) were mainly based on data from four large randomized trials initiated in the 1980s and 1990s, reporting an AAA prevalence of 3.9–7.7% in the male population > 65 years of age [18, 22, 27, 28]. Based on these data, the USA, the United Kingdom (UK) and Sweden implemented national screening programs, with Germany establishing theirs in 2018 [8]. According to the national guidelines in Germany, every man older than 65 years is entitled to a free ultrasound examination of the abdominal aorta. Apart from the above-mentioned countries, there are ongoing trials, such as in New Zealand and Italy [12, 16, 21, 30], supporting screening at-risk populations and also women, in the absence of a formal screening program. Present guidelines also support focused screening of subjects with a distinct risk profile [6, 11], as AAA is associated with cardiovascular risk factors, cardiovascular and pulmonary diseases (e.g. COPD), male gender, smoking and age [2, 33, 34].

Screening for AAA has been proven as an effective preventive health measure, which is cost-effective [35, 36], though nowadays faced to a declining prevalence of 1–2% and a decreased incidence of ruptured AAAs in Western countries [1, 5, 7, 25, 31].

Against this background, the present study evaluated the prevalence of abdominal aortic aneurysms in a male high-risk cohort (≥ 65 years of age), hospitalized for suspected or known cardiopulmonary disease. Furthermore, we used vascular ultrasound expertise to determine the co-prevalence of associated lower extremity arterial aneurysms.

## Methods

### Study design

By resolution of the GBA (Federal Joint Committee of Insurance Companies and Physicians) of March 2017 and renumeration by statuatory health insurances [26], a nationwide screening program for AAA was established in Germany, starting on January 1, 2018. This study was conducted as direct comparison to the current screening program, to investigate potential benefits of screening a high-risk cohort in an in-hospital setting.

### Setting

This observational study was performed in the First Department of Medicine, University Medical Centre Mannheim, Germany, which is an universitary hospital located in an urban area with a population of over 300.000 inhabitants. Study enrolment started on January 1, 2018 over one year until January 2019. Patients were not followed up.

The study complies with the Declaration of Helsinki and was approved by the local ethical committee, Medical Ethic Commission II, Faculty of Medicine Mannheim, University of Heidelberg, Germany. All patients gave verbal consent to the ultrasound screening and data collection. Data were analyzed anonymously. As the ultrasound examination is part of the clinical routine in cardiovascular medicine without known harmful effects, verbal consent was considered sufficient and approved by the local ethics committee. Data protection was in accordance with the EU Data Protection Directive.

### Participants

Five hundred sixty-six male inpatients (≥ 65 years of age), hospitalized for suspected or known cardiopulmonary disease were enrolled. Patients with advanced oncological concomitant diseases and limited survival rates were excluded. In case of uncertain mental condition or suspicion of low survival rates geriatric assessment was performed.

### Variables

#### Data collection

Data were collected in pseudonymized form. Comorbidities were recorded based on medical history, such as hypertension, hyperlipidaemia, diabetes mellitus and history of smoking. The diagnosis of coronary artery disease (CAD) was proven angiographically. Chronic kidney disease (CKD) was defined as glomerular filtration rate (GFR) < 60 ml/min. The main symptoms leading to hospital admission and the definitive diagnosis of the hospital stay were recorded from the discharge papers, as well as concomitant medication at discharge.

#### Laboratory parameters

Blood samples were collected under fasting conditions to determine total cholesterol and LDL-cholesterol.

#### Imaging and measurements

Abdominal aortic ultrasound scans were performed by two experienced angiologists (HM and MS) under fasting conditions. Using a high-end ultrasound scanner (Mindray, Resona 7) with a convex 3.5 MHz transducer, the infrarenal aorta was visualized in its entire length in transverse and longitudinal views. The maximum diameter was measured using the leading edge to leading edge (LELE) method in the plane perpendicular to the arterial axis [15]. AAA was defined by a diameter of ≥30 mm. During the examination vital parameters (heart rate, blood pressure) were recorded. Screening for suprarenal aortic aneurysms was not performed. Upon detection of an AAA (LELE ≥30 mm), the lower extremity arteries were examined for the presence of iliac, femoral, or popliteal aneurysms. All patients which were newly diagnosed with an AAA were treated according to the present ESC guidelines on the diagnosis and treatment of aortic diseases [11].

#### Reproducibility

Prior to patient enrollment a cohort of 40 volunteers was examined by both angiologists (HM and MS) for validation. Inter-observer agreement was determined using Cohen’s kappa statistics.

### Study size

A sample size calculation was performed. Assuming an all-over prevalence of ca. 1% in the population with assuming double the proportion in a high-risk cohort, the estimated sample size would be 474. All patients meeting the inclusion criteria were systematically screened, leading to a number of 614 study participants, whereof 34 were excluded after geriatric assessment, due to uncertain mental condition or suspicion of low survival rates.

### Statistical analysis

Data are presented as mean ± standard deviation, median (interquartile range), or frequency (percentage). Continuous variables were compared using a two-tailed Student’s *t*-test for parametric and Mann–Whitney *U* test for non-parametric variables. Categorical variables were compared with the *χ*^2^ test. Inter-observer agreement for abdominal aortic ultrasound was determined using Cohen’s kappa statistics, providing a coefficient ranging from 0 to 1 and its 95 CI, with a high value close to 1 indicating high similarity. Multivariate analysis was performed with logistic regression analysis using block entry of the following variables: history of smoking, diabetes mellitus and coronary 3-VD, provided to have a *p* <  0.01 in univariate analysis.

For all statistical analyses, *p* <  0.05 was considered statistically significant. All statistical analyses were performed using Statistical 1 Package for Social Sciences (SPSS version 23, IBM, Munich, Germany).

## Results

### Patients’ characteristics

Six hundred fourteen patients were eligible for inclusion. Thirty-four patients were excluded due to estimated low survival rates (< 2 years according to geriatric assessment). Fourteen patients were not examined, either because their mental or physical states were considered inadequate, or because they refused the examination after they had been verbally informed regarding possible benefits (early detection) and detriments (anxiety, indication for intervention, risk of intervention). A total of 566 male patients (≥ 65 years of age) was examined. Their median age was 78.0 [72.0–82.0] years. The patients’ characteristics are shown in Table 1.

**Table 1:** Patients’ characteristics according to absence or presence of AAA

	All patients (*n* = 566)	No AAA (*n* = 526, 92.9%)	AAA ≥ 30 mm (*n* = 40, 7.1%)	*p*-value
Age (median, IQR)	78.0 [72.0–82.0]	77.0 [72.0–82.0]	78.5 [74.8–83.0]	0.41
Heart rate (bpm)	74.9 ± 14.6	75.0 ± 14.8	73.4 ± 12.0	0.51
RR sys (mmHg)	140.3 ± 19.3	140.5 ± 19.2	138.0 ± 20.7	0.43
RR dia (mmHg)	80.4 ± 11.1	80.5 ± 11.2	79.1 ± 10.8	0.44
Total cholesterol (mg/dl)	132.1 ± 43.9	131.9 ± 43.8	137.2 ± 49.7	0.72
LDL (mg/dl)	91.7 ± 32.7	91.8 ± 32.7	88.0 ± 32.9	0.73
*Comorbidities, n (%)*				
Hyperlipidemia	370 (65.4)	339 (64.4)	31 (77.5)	0.11
Hypertension	470 (83.0)	433 (82.3)	37 (92.5)	0.10
History of smoking	327 (57.8)	294 (55.9)	33 (82.5)	0.01
Diabetes mellitus	251 (44.3)	240 (45.6)	11 (27.5)	0.03
CAD	393 (69.4)	360 (68.4)	33 (82.5)	0.01
Coronary 3-vessel-disease	194 (34.3)	168 (31.9)	26 (65.0)	< 0.0001
Chronic kidney disease (GFR < 60 ml/min)	340 (60.1)	311 (59.1)	29 (72.5)	0.14
*Main diagnosis, n (%)*				
ACS	246 (43.5)	224 (42.6)	22 (55.0)	0.13
Heart failure	182 (32.2)	174 (33.1)	8 (20.0)	0.09
Exacerbation of COPD/ Pneumonia	68 (12.0)	63 (12.0)	5 (12.5)	0.92
Others	31 (5.5)	28 (5.3)	3 (7.5)	0.56
*Main symptoms, leading to hospital admission, n (%)*				
Angina pectoris	166 (29.3)	155 (29.5)	11 (27.5)	0.79
Dyspnea	208 (36.7)	189 (35.9)	19 (47.5)	0.14
Palpitations/ Syncope	104 (18.4)	99 (18.8)	5 (12.5)	0.32
Others	35 (6.2)	34 (6.5)	1 (2.5)	0.32
*Concomitant medication, n (%)*				
Betablocker	331 (58.5)	308 (58.6)	23 (57.5)	0.90
Calcium channel blocker	100 (17.7)	96 (18.3)	4 (10.0)	0.19
ACE inhibitor/ ARB	444 (78.4)	411 (78.1)	33 (82.5)	0.52
Statin	375 (66.3)	339 (64.5)	36 (90.0)	0.001
Antiplatelet therapy	312 (55.1)	284 (54.0)	28 (70.0)	< 0.05
VKA/ DOAC	190 (33.6)	177 (33.7)	13 (32.5)	0.88

*CAD* coronary artery disease. *GFR* glomerular filtration rate. *ACS* acute coronary syndrome. *COPD* chronic obstructive pulmonary disease. *IQR* interquartile range

The three most frequent main symptoms leading to hospital admission were angina pectoris, dyspnea, and palpitations/syncope. The three main diagnoses at the time of hospital discharge were acute coronary syndrome (43.3%), congestive heart failure (32.2%), or chronic pulmonary disease with or without pneumonia (12.0%). Comorbidities of the overall cohort included angiographically verified coronary artery disease (CAD, 69.4%), arterial hypertension (82.9%), diabetes mellitus (44.4%), and 57.6% had a history of smoking.

### Findings of the infrarenal aorta

Visualization of the abdominal aorta was possible in all patients, although a ‘second-look’.

ultrasound was required in 3 patients during the hospital stay to obtain adequate measurements. AAAs were detected in 40 out of 566 patients, yielding an overall prevalence of 7.1%. Inter-observer agreement was determined using Cohen’s kappa statistics, with a value of 0.98 [0.93 to 1.0], indicating nearly perfect agreement.

The frequencies of previously undetected, already diagnosed, and previously (endovascular or open-surgically) repaired AAAs, as well as the distribution of their sizes (small, medium, or large) are presented in Table 2.

**Table 2:** Distribution of newly screen-detected, previously diagnosed and previously repaired infrarenal AAA according to the AAA size

	Newly screen-detected AAA (*n* = 14, 2.5%)	Previously diagnosed AAA (*n* = 14, 2.5%)	Previously repaired AAA (*n* = 12, 2.1%)
*Max. Aortic diameter (mm)*^*a*^			
30—44	12	10	0
45—54	2	4	2^b^
≥ 55	0	0	10^b^

AAA abdominal aortic aneurysm

^a^The diameter was measured using the leading edge-to-leading edge (LELE) method

^b^Documented values before the aortic repair were specified

### Associations with AAA

Predictors associated with the presence of AAA in the patients’ cohort can be found in Table 3. Univariate analysis revealed an increased prevalence of smoking history (*p* <  0.001) and coronary three-vessel-disease (3-VD) (*p* <  0.0001) and a decreased prevalence of diabetes mellitus (*p* <  0.01). Neither hyperlipidemia (*p* = 0.110), nor arterial hypertension (*p* = 0.099) or chronic kidney disease (*p* = 0.138) were associated with AAA.

**Table 3:** Predictors associated with the presence of AAA in the patients’ cohort

Predictor	Univariate	*p*-value	Multivariate	*p*-value
	OR (95% CI)		OR (95% CI)	
History of smoking	−0.13 (− 0.11 to − 0.03)	< 0.001	3.7 (1.6 to 8.6)	< 0.01
Diabetes mellitus	0.12 (0.02 to 0.10)	< 0.01	0.5 (0.2 to 0.9)	0.03
Coronary 3-VD	−0.18 (− 0.15 to − 0.05)	< 0.0001	4.5 (2.3 to 8.9)	< 0.0001

*3-VD* three vessel disease. *CI* confidence interval

Based on the results of the univariate analysis, the following variables were included in the multivariate analysis, which revealed as independent predictors: coronary 3-VD (OR: 4.5, CI: 2.3–8.9, *p* <  0.0001) and a history of smoking (OR: 3.7, CI: 1.6–8.6, *p* <  0.01) were positively associated with AAA, while diabetes mellitus (OR: 0.5, CI: 0.2–0.9, *p* = 0.0295) showed a negative association with the presence of AAA.

### Associated aneurysms

Among 40 patients with AAA, we found four patients with previously unknown large aneurysms of the lower extremity arteries: two with aneurysms of the common iliac artery > 30 mm, and two with asymptomatic popliteal aneurysms > 20 mm and poor crural vessel runoff, suggestive of a previous embolism.

## Discussion

Current national population-based screening programs for AAA of all men at or over 65 years have been challenged as the effect of the screening program might be smaller than initially calculated. Therefore we tried to ascertain if focused screening in a high-risk cohort may be more effective.

The main findings of the present study are:

I. The overall prevalence of AAA (> 30 mm) in 566 patients hospitalized for known or suspected cardiopulmonary disease was considerably high (40 patients, 7.1%), which can be subdivided into
II. moderate new diagnoses (14 patients, 2.5%) of AAA, none of which was large (> 55 mm), already diagnosed (14 patients, 2.5%) or previously repaired AAA (12 patients, 2.1%).

With Germany establishing the national screening program in 2018, this study was designed as direct comparison, investigating especially the prevalence of AAA in an hospitalized high-risk cohort compared with the general population. A significantly decreasing prevalence of 1–2% in Western countries in 65-year-old men has been described [7, 32], compared to 3.5% in the Viborg trial at that age [14]. In our high-risk cohort, we found an overall prevalence of 7.1%, which is comparable with studies in France and Belgium in a similiar setting [9, 19].

Following the current German national program for AAA screening, we screened only male inpatients aged ≥65 years. Gianfagna et al. evaluated the AAA prevalence in the general population and subgroups at high cardiovascular risk, including also women and younger subjects [12], which detected an overall prevalence of 1.9% and peaks of more than 4% in patients with previous myocardial infarction and ever smokers. In this cross-sectional study, patients were randomly selected for ultrasound examination. It would have been interesting to examine also younger men and women in our study, within the in-hospital setting. Even though screening a high-risk population will detect a higher AAA prevalence, it might also detect patients with a lower overall survival and limited life expectancy, reflected by the median age of 78.0 years in our population, and thus questionable benefit. Therefore, targeted screening, also in younger subgroups at higher risk merit evaluation.

AAA and atherothrombotic diseases often coincide, presumably due to related cardiovascular risk factors [29]. In accordance to recent studies we found a strong association between AAA and CAD [10, 20]. In our study, the subgroup of men with angiography-verified CAD revealed an AAA prevalence of 8.4 and 13.4% among those with three-vessel CAD. Smoking was also found as independent predictor, which is known as the most important modifiable risk factor [16]. Furthermore, we could confirm a “protective effect” of Diabetes mellitus [3, 13], which might be explained on one hand by the intensive treatment of patients with diabetes mellitus and a distinct risk profile [24] and on the other may glycated cross-links in the aorta play a protective role [3]. These observations were also made in the REACH Registry [4], which showed a strong association of AAA with a history of smoking and an inverse relation with diabetes. Similiar to the RoCAV population based study [12] the AAA prevalence was evaluated in out-patients with a cardiovascular risk profile. These studies both showed lower prevalences for AAA (2.5% in the REACH Registry [4] and 1.9% in the italian population [12]) than in our cohort, which can be explained by the inclusion criteria and the setting. First, the mean age of these study populations was less than 70 years compared with our population with a median age of 78.0 years, as younger patients (< 65 years) were included. The mentioned studies report the prevalence of AAA in out-patients. Screening in-hospital patients could target at-risk individuals who do not participate in national out-patient screening programs. Moreover, women were also included in these two studies. As we conducted our study as direct comparison to the national screening program, we adopted the same criteria, with excluding women. It would have been interesting to screen also hospitalized women, as females have worse prognosis than men and are older at the time of the first diagnosis [17, 23].

The moderate prevalence of 2.5% of previously unknown AAA must be seen in the context of the following: the majority of the patients were in frequent contact with the health system before the index hospital admission. AAAs may be detected as incidental finding within routine diagnostic abdominal imaging. Furthermore, the implementation of population-based screening programs is supposed to decrease the in-hospital detection rates of previously unknown AAAs. In our cohort, 14 patients had a first diagnosis of AAA, which is approximately double the proportion of AAAs in current population-based screening programs of Western countries. We did not find any large AAAs (> 55 mm) among the newly-screen detected.

Furthermore, we performed additional screening for associated aneurysms of the lower extremity arteries in all patients with an AAA, independent of the time of the diagnosis. Among 40 patients with AAAs, 4 patients had previously unknown large aneurysms of their lower extremity arteries, yielding a prevalence of 10%, which is in accordance to the literature (37), with expecting even higher prevalence in the entire study cohort, though systematic screening has not been performed.

Our study supports the usefulness of screening subgroups at high cardiovascular risk. However, as patients were not followed, a cost-effectiveness evaluation was not performed, which limited the interpretation of the results.

### Strengths and limitations of this study

- Ultrasound screening for AAA was performed in a high-risk inpatient cohort (men > 65 years of age) using the selection criteria of current population-based national screening programs.
- In those patients admitted to our institution, we identified AAA that were previously unknown, diagnosed, or repaired.
- Furthermore, all patients with any AAAs were systematically screened for AAA-associated lower extremity arterial aneurysms.
- The lack of long-term follow-ups and cost-effectiveness evaluations limited the interpretation of the results and does not contribute to the benefit-versus-harm discussion in AAA screening.

## Conclusion

Ultrasound screening in male inpatients, hospitalized for suspected or known cardiopulmonary disease, revealed a high AAA prevalence AAA in comparison to the present epidemiological screening programs. Furthermore, additional systematically screening of the lower extremity arteries yielded a relevant number of associated aneurysms with indication for possible intervention.

Performing AAA screening in a cardiopulmonary inpatient cohort combines high prevalence of cardiovascular risk factors and vascular ultrasound expertise. Screening this high-risk cohort for AAA and associated aneurysm would be feasible.

## Data Availability

Datasets are available from the corresponding author upon reasonable request.

## Abbreviations

3-VD: Three-vessel-disease
AAA: Abdominal aortic aneurysm
ACS: Acute coronary syndrome
CAD: Coronary artery disease
CI: Confidence interval
COPD: Chronic obstructive pulmonary disease
GFR: Glomerular filtration rate
IQR: Interquartile range
LELE: Leading edge to leading edge
PAA: Popliteal artery aneurysm
